# Local arterial administration of acidified malonate as an adjunct therapy to mechanical thrombectomy in ischemic stroke

**DOI:** 10.1093/cvr/cvaf118

**Published:** 2025-06-27

**Authors:** Jordan J Lee, Hiran A Prag, Karthik Chary, Jiro Abe, Shinpei Uno, Annabel Sorby-Adams, Chak Shun Yu, Olga Sauchanka, Amin Mottahedin, Joshua D Kaggie, Ferdia A Gallagher, Michael P Murphy, Thomas Krieg

**Affiliations:** Department of Medicine, University of Cambridge, Addenbrookes Hospital, Hills Road, Cambridge CB2 0QQ, UK; Department of Medicine, University of Cambridge, Addenbrookes Hospital, Hills Road, Cambridge CB2 0QQ, UK; Preclinical Imaging Suite, Anne McLaren Building, University of Cambridge, CB2 0BA, UK; Department of Radiology, University of Cambridge, Addenbrookes Hospital, Hills Road, Cambridge CB2 0QQ, UK; Department of Medicine, University of Cambridge, Addenbrookes Hospital, Hills Road, Cambridge CB2 0QQ, UK; MRC Mitochondrial Biology Unit, University of Cambridge, Hills Road, Cambridge CB2 0XY, UK; Department of Neurology and the Center for Genomic Medicine, Massachusetts General Hospital and Harvard Medical School, Boston, Massachusetts 02114, USA; MRC Mitochondrial Biology Unit, University of Cambridge, Hills Road, Cambridge CB2 0XY, UK; Department of Medicine, University of Cambridge, Addenbrookes Hospital, Hills Road, Cambridge CB2 0QQ, UK; Nuffield Department of Clinical Neurosciences, University of Oxford, John Radcliffe Hospital, Oxford OX3 9DU, UK; Preclinical Imaging Suite, Anne McLaren Building, University of Cambridge, CB2 0BA, UK; Department of Radiology, University of Cambridge, Addenbrookes Hospital, Hills Road, Cambridge CB2 0QQ, UK; Preclinical Imaging Suite, Anne McLaren Building, University of Cambridge, CB2 0BA, UK; Department of Radiology, University of Cambridge, Addenbrookes Hospital, Hills Road, Cambridge CB2 0QQ, UK; Department of Medicine, University of Cambridge, Addenbrookes Hospital, Hills Road, Cambridge CB2 0QQ, UK; MRC Mitochondrial Biology Unit, University of Cambridge, Hills Road, Cambridge CB2 0XY, UK; Department of Medicine, University of Cambridge, Addenbrookes Hospital, Hills Road, Cambridge CB2 0QQ, UK

**Keywords:** Ischaemia/reperfusion injury, Stroke, Mechanical thrombectomy, MCT1 transporter, Mitochondria, Malonate

## Abstract

**Aims:**

Ischemic stroke is increasingly treated by mechanical thrombectomy (MT) with the more rapid and complete reperfusion of the ischemic tissue, enhancing patient outcome, compared to recombinant tissue plasminogen activator (rtPA) alone. Even so, there is still extensive brain infarction and disability following MT, which is exacerbated by ischemia–reperfusion injury (IRI) and other pathological processes during reperfusion. Hence, an adjunct therapy to MT that decreases IRI should enhance patient outcomes.

**Methods and results:**

To test this possibility, we adapted the transient middle cerebral artery occlusion (tMCAO) mouse model to allow local intra-arterial administration of acidified disodium malonate (aDSM) to decrease IRI as the ischemic tissue was reperfused. Administration of aDSM (160 mg/kg; pH 6) during reperfusion decreased brain infarct volume by ∼60% when assessed by magnetic resonance imaging (MRI) 24 h after reperfusion and improved neurological function.

**Conclusion:**

These findings suggest aDSM as a potential adjunct therapy to further improve outcomes for stroke patients treated by MT.

## Graphical Abstract

**Graphical Abstract:**
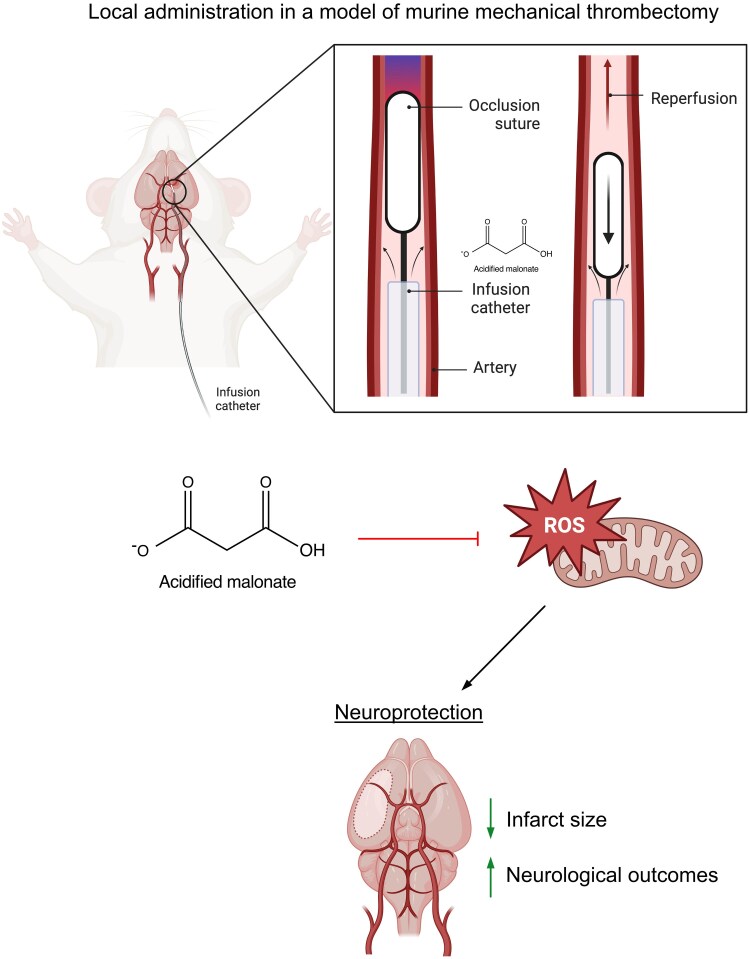

**Time of primary review: 110 days**

## 1. Introduction

**Figure 1:**
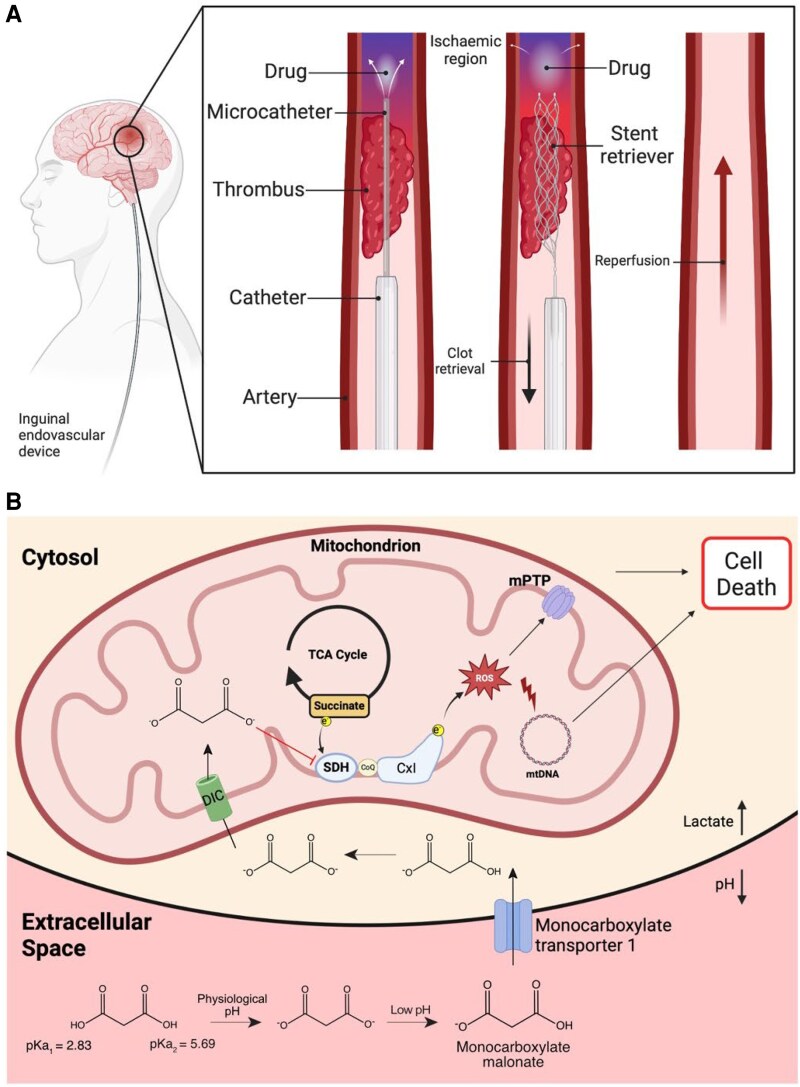
Schematic background to proposed adjunct treatment of ischemic stroke. *A*: Proposed adjunct treatment to be used with human mechanical thrombectomy to prevent IRI. *B*: Mechanism of action of acidified malonate (aDSM) in preventing IRI. DSM administered locally post the blood clot at pH 6 leads to the formation of monoprotonated malonate, due to its relatively high pKa, which is then rapidly taken up through the BBB via MCT1. Within the cell, malonate is then taken up into mitochondria via the mitochondrial dicarboxylate carrier (DIC) in the mitochondrial inner membrane. Within the mitochondrial matrix, the malonate then acts as a competitive inhibitor of SDH, preventing the ROS production at complex I by RET.

**Figure 2:**
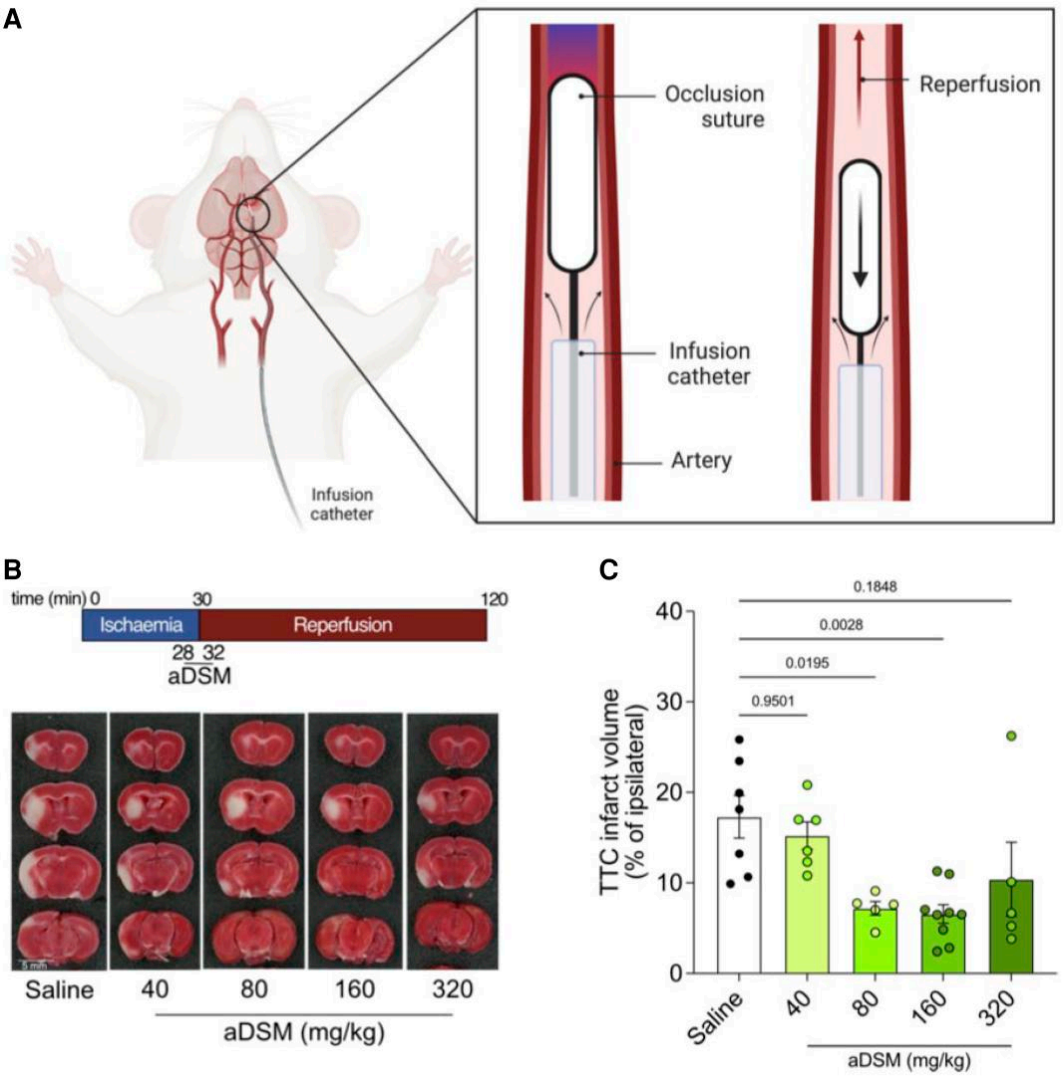
Intra-arterial infusion of acidified malonate is protective against stroke IRI following tMCAO, measured at 2 h. a: The occlusion suture is first inserted into the internal carotid artery (CA) and advanced to the MCA to bring about MCAO, as verified by measuring cerebral blood flow (CBF) by laser Doppler flowmetry. Shortly prior to reperfusion, the infusion catheter is then inserted into the CA on top of the occlusion suture and guided to the region of occlusion/ischemia. Withdrawal of the occlusion suture from the MCA back to the CA initiates reperfusion. The infusion catheter is used to infuse 50 µL saline or drug from 2 min before to 2 min after the onset of reperfusion at the site of ischemia. *B*: Representative TTC-stained brain slices assessed 2 h after reperfusion with either saline or indicated doses of aDSM. *C*: Dose response of brain infarct volume to aDSM assessed by TTC staining 2 h after reperfusion. Data are mean infarct volume ± SEM, *n* = 5–9. Statistics: One-way ANOVA with Tukey’s *post hoc* test.

Current acute treatment for ischemic large- and medium-vessel stroke aims to achieve timely recanalization of the occluded artery either pharmacologically with thrombolytics, or increasingly by mechanical thrombectomy (MT)^1^ (*Figure 1A*). MT is used to treat large vessel occlusions and improves outcomes.^1–8^ Even so, rapid reperfusion of ischemic tissue following MT leads to ischemia–reperfusion injury (IRI) and secondary brain injury.^9,10^ There are no approved treatments for IRI, but recent developments offer translational opportunities.^11^ During ischemia, succinate accumulates in tissues, including the brain.^12,13^ Upon reperfusion, this succinate is rapidly oxidized by succinate dehydrogenase (SDH), driving the production of reactive oxygen species (ROS) by reverse electron transport (RET) at complex I that initiates IRI.^12–15^ Inhibiting SDH upon reperfusion with disodium malonate (DSM) reduces tissue infarction, and the potency of DSM is greatly enhanced by acidification due to the rapid cell uptake of protonated malonate via the monocarboxylate transporter 1 (MCT1).^12,16^ Thus, the administration of aDSM is a potential therapy to attenuate IRI (*Figure 1B*). However, as succinate is very rapidly oxidized upon reperfusion, to be effective, aDSM must be present in the tissue at the onset of reperfusion to be protective.^16^ The development of MT enables this key limitation to be overcome in stroke patients by infusing aDSM via an intra-arterial microcatheter into the ischemic zone prior to clot retrieval (*Figure 1A*). To assess the potential of this approach, we developed a mouse middle cerebral artery occlusion (tMCAO) model of MT incorporating an intra-arterial catheter to deliver aDSM via the internal carotid artery (ICA) upon reperfusion following 30-min ischemia (*Figure 2A*). A range of endpoints, including clinically relevant magnetic resonance imaging (MRI) and neurological scoring, were then used to assess the efficacy of aDSM at decreasing IRI. These findings support the local and timely infusion of aDSM in conjunction with MT to improve the outcomes for stroke patients.

## 2. Methods

### 2.1 Animals

Animal procedures were carried out in accordance with the UK Animals (Scientific Procedures) Act of 1986 and the University of Cambridge Animal Welfare Policy. Procedures were reviewed by the University of Cambridge Animal Welfare Ethical Review Board and approved to be carried out under the Project License PP4344323.

Male C57BL/6J and BKS.Cg-Dock7^m^ +/+ Lepr^db^/J mice were ordered from Charles River Laboratories UK (Margate, UK). The ND6-P25L mouse strain was generously provided by Professor Douglas Wallace, University of Pennsylvania, and was backcrossed onto the C57BL/6J background. All mice were housed in a room on a 12 h light/dark cycle with *ad libitum* access to chow and water. Mice were acclimatized in our facility for a week before being used in the experiments. At the time of experiment, young adult mice were aged 8–12 weeks and weighed 24–30 g, aged mice were aged 55–57 weeks and weighed 30–38 g, ND6-P25L mice were aged 20 weeks and weighed 30.2–33.2 g, whilst db/db mice were aged 8 weeks and weighed 33.4–38.9 g.

### 2.2 Murine transient middle cerebral artery occlusion (tMCAO) stroke model

The middle cerebral artery (MCA) was occluded to induce a stroke in an acute *in situ* mouse model as previously described^17^ to assess the effects of malonate on stroke IRI. Briefly, mice were anaesthetized with isoflurane (3% induction, 1.5–2% maintenance) given at 1 L/min O_2_ using TEC 3 Isoflurane Vaporiser (98723N, +Mediquip LTD). A Doppler flowmetry probe (PeriFlux System 5000, Perimed, Sweden) was attached to the ipsilateral side of the skull to monitor and record blood flow in the MCA territory. A midline incision on the neck was made to expose the left common carotid artery (CCA) and external carotid artery, where both were permanently ligated. Then, the left ICA was temporarily clamped before an incision was made, allowing a 6–0 suture with a silicone-coated tip (602223PK10RE, Doccol, USA) to be inserted. Once inserted, the vascular clamp was removed, and the suture was advanced toward the MCA. MCAO was confirmed when a drop in blood flow was observed on the Doppler, denoting the start of ischemia. The suture was left *in situ*. Animals that did not have *a* > 70% drop in cerebral blood flow in relation to the baseline were excluded from the study. After 30 min of ischemia, the suture was removed, allowing reperfusion. For ischemic measurements of metabolites, the suture was left *in situ* to prevent reperfusion. Mice were sacrificed by cervical dislocation, and brains were collected for triphenyltetrazolium chloride (TTC) infarct measurements. For malonate analysis, mice were sacrificed by cervical dislocation and flushed by perfusing ice-cold saline through the heart before snap-freezing in isopentane. For oxidative damage and succinate analysis, mice were sacrificed by cervical dislocation, and the ipsilateral and contralateral hemispheres were quickly dissected and snap-frozen in isopentane.

### 2.3 Local intra-arterial infusion of aDSM in conjunction with tMCAO

To allow for local infusion at the site of ischemia, a thin polyimide catheter (Zeus PN: 270903, Polyimide; Tubing; internal diameter: 172 ± 12 µm; outside diameter: 216 ± 12 µm) was inserted on top of the 6-0 occlusion suture and advanced toward the MCA. Infusion was started 2 min before and continued 2 min after reperfusion. The occlusion suture/catheter was retracted together to initiate reperfusion, confirmed by laser Doppler flowmetry, and remained *in situ* for the duration of the infusion.

### 2.4 TTC staining

Following craniotomy, the isolated brains were sliced coronally into five 2-mm slices in a brain matrix (Zivic Instruments) and incubated in 2% TTC, Sigma-Aldrich) for 8 min at 37°C. Brain slices were then fixed in paraformaldehyde (PFA) at 4°C until imaging. Image J software (version 1.48)^18^ software was used to delineate the red viable tissue and the white infarcted tissue in the ipsilateral and contralateral hemispheres. Infarct volume was calculated as % ipsilateral volume, i.e. infarct volume over the whole ipsilateral hemisphere volume.

### 2.5 *In vivo* magnetic resonance imaging

*In vivo* MRI was performed using a 3T Bruker BioSpec MRI system with a 1H transmit-receive mouse head volume coil with an inner diameter of 23 mm. The mice were first anaethetized with 3% isoflurane and then maintained under 1–2% isoflurane during the scan. Respiration rate and temperature were monitored using a respiration pneumatic sensor and a rectal temperature probe, and their physiologic stability was maintained (respiration rate at 30–50 bpm, temperature at 35–37°C) with a water circulation system (ThermoFisher Scientific).

The MRI sequence parameters are listed in Supplementary Data Supplementary material online, *Table S1*. Initial scout scans were performed using a single- and multi-slice localizer to ensure sufficient brain coverage for the subsequent scans. Anatomical images were acquired using high-resolution T1-weighted rapid acquisition relaxation enhancement (RARE) inversion recovery (IR) and T2-weighted Turbo RARE sequences. Diffusion-weighted contrast images were acquired using a diffusion-weighted spin-echo (DWI-SE) sequence. For apparent diffusion coefficient (ADC) mapping, a 2D spin-echo echo-planar imaging (SE-EPI) sequence was used.

### 2.6 MRI data analysis

A mono-exponential signal decay model was used to estimate ADC values using the image sequence analysis tool in ParaVision 360 (v3.3). The anatomical T1w and T2w images were converted to NIfTI format for analysis. Infarct volumes on T2w images and DWI (b1200 s/mm^2^) were performed via active contour segmentation in ITK-SNAP^19^ (version 3.8.0). Midline shift was measured as a surrogate marker of cerebral edema using the FMRIB Software Library (FSL)^20,21^ and ITK-SNAP. T2w images were manually masked and brains extracted to facilitate linear affine registration to a murine atlas.^22^ Once registered, the distance in mm from the midline of the paraventricular nucleus at the level of the third ventricle was quantified.

### 2.7 Assessment of neurological outcome after tMCAO

The Bederson (0–5) score, as described previously by Bieber *et al*.^23,^ was used to assess neurological function in mice 24 h after tMCAO. The Bederson scores are as follows: 0, no observable deficit; 1, forelimb flexion; 2, forelimb flexion and decreased resistance to lateral push; 3, circling; 4, circling and spinning around the cranial-caudal axis; and 5, no spontaneous movement. To carry out this assessment, mice were allowed to move around freely for an initial observation, where circling behavior was assessed. After which, the animal was held by the tail mid-air and observed for forelimb flexion. Thereafter, the animal was placed on a surface where lateral pressure was applied on each side of the animal, registering sliding behavior to assess resistance to lateral push.

### 2.8 Assessing rtPA protease activity in vitro

We assessed the activity of recombinant tissue plasminogen activator (rtPA) using an AnaSpec Sensolyte AMC rtPA activity end point fluorometric assay kit in 96-well format. The effect of a range of concentrations of neutral DSM on this activity was assessed with the rtPA inhibitor Leupeptin used as a negative control. In all cases, DSM (neutral) or aDSM (pH 6) was diluted to 10*×* final concentration in H_2_O and 10 µL added to each relevant well in triplicate. Leupeptin was used at a final concentration of 1 mM. rtPA fluorescent substrate was diluted in assay buffer (proprietary, pH 8.5) and added at a final concentration of 10 µM substrate. rtPA diluted in assay buffer was added to relevant wells at 5 U/well. The microplate was protected from light and incubated at RT for 30 min, and then read on a fluorescent plate reader (λ_Ex_ = 354 nm, (λ_Em_ = 442 nm).

### 2.9 LC-MS/MS analysis of malonate and succinate

Malonate and succinate were extracted from tissues and analyzed by LC-MS/MS as previously described.^24–26^ LC-MS/MS Analysis of malonate and succinate was performed using an LCMS-8060 mass spectrometer (Shimadzu, UK) with a Nexera X2 UHPLC system (Shimadzu, UK). The separation of metabolites was achieved by using a SeQuant ZIC-HILIC column (3.5 μm, 100 Å, 150 *×* 2.1 mm, 30°C column temperature; Merck Millipore, UK) with a ZIC-HILIC guard column (200 Å, 1 × 5 mm) at a flow rate of 200 μL/min with mobile phases of (i) 10 mM ammonium bicarbonate and (ii) 100% acetonitrile. The mass spectrometer was operated in negative ion mode with multiple reaction monitoring (MRM), and spectra were acquired using Labsolutions software (Shimadzu, UK). Malonate and succinate were quantified using standard curves relative to 1 nmol of [^13^C_3_]-malonate and [^13^C_4_]-succinate internal standard, respectively.

### 2.10 Malondialdehyde assay

Lipid peroxidation was determined by measuring the levels of malondialdehyde using the TBARS Assay Kit (ab118970, Abcam) according to the manufacturer’s instructions. Briefly, ∼25 mg of frozen brain tissue was weighed into cooled lysis tubes (Precellys, CK-14). 150 µL of ddH_2_O and 3 µL of butylated hydroxytoluene (BHT) stock solution were added to the samples. Then 150 µL of 2N perchloric acid was added and the mixture was homogenized in a bullet blender (Bertin Instruments, Precellys 24) at setting 6500 for 15 s (CK-14 beads). After centrifugation, 200 µL of supernatant was collected and mixed with 600 µL TBA reagent, which was incubated at 95 ^o^C for 60 min. After the samples were cooled on ice, the fluorescence intensity was measured using a fluorometric plate reader (Molecular Devices Spectra Max Gemini XS, λ_Ex_ = 515 nm, λ_Em_ = 553 nm). One of the measurements comparing Malondialdehyde (MDA) levels in the ipsilateral and contralateral hemispheres following IRI from saline-treated mice gave a ratio of MDA levels in the ipsilateral and contralateral hemispheres of 7.3, which is 5.3-fold greater than the mean ratio of 1.369 ± 0.198 for the other mice. Thus, this data set was identified as an outlier and omitted from subsequent analysis. This does not impact the conclusions of the experiments shown in *Figure 4D* and Supplementary Data Supplementary material online, Figure*S5C*.

### 2.11 Complex I activity

Complex I activity from tissues was assessed as described in detail previously.^27^ To prepare samples for complex I activity and citrate synthase activity assays, 5 mg of tissue was placed into pre-chilled lysis tubes (CK14; Precellys, Bertin Instruments, France) and 400 µL ice-cold 50 mM KPi buffer (pH 7.8) added. Samples were homogenized using a Precellys 24 homogenizer (6500 rpm, 15 s; Bertin Instruments, France). Protein concentration of the homogenate was measured by bicinchoninic acid (BCA) assay, samples flash frozen, and stored at −70°C until assays were carried out.

Tissue homogenate was thawed on ice just prior to the assay and diluted to 0.1 mg/mL with 50 mM KPi buffer supplemented with 0.05% decylubiquinone (DDM) and achieving a final concentration of 0.025% DDM. Complex I activity was carried out by monitoring the decrease in NADH levels due to its oxidation, which occurs when it encounters the electron acceptor decylubiquinone (dQ) in the test environment.^28,29^ This process is sensitive to the presence of the specific complex I inhibitor rotenone. Antimycin A and potassium cyanide (KCN) were included to prevent electrons from passing beyond complex I in the respiratory chain. The change in NADH concentration was observed via a dual-wavelength UV–Visible spectroscopy method, using a microplate reader (SPECTRAmax Plus 384, Molecular Devices, UK). The experimental setup used 96-well plates, each with an assay volume of 200 µL. In a 96-well plate, 50 µL assay buffer (50 mM KPi supplemented with 0.2 mM KCN, 0.3 µM antimycin A, 100 µM dQ ± 0.5 µM rotenone; pH 7.8) was added to each well and the plate kept on ice. 100 µL of diluted homogenate was added prior to initiating the reaction with 50 µL NADH, achieving a final concentration of 0.2 mM. The plate was read over 30 min at 30°C, and NADH oxidation was tracked by measuring the absorbance at wavelengths of 340 and 380 nm, at intervals of 8–12 s. To ascertain the maximum linear rate of NADH oxidation, the absorbance difference between 340 and 380 nm for at least duplicate samples was computed. This rate was then corrected by removing the rotenone-insensitive background rate, which was obtained from samples containing rotenone. The concentration of NADH was determined by applying the extinction coefficient (ε340–380) of 4.81 mM-1cm-1.

### 2.12 Citrate synthase activity assay

Citrate synthase activity was measured using the enzyme-induced conversion of acetyl-CoA and oxaloacetate into citrate, which simultaneously generates CoA.^30,31^The presence of CoA was detected using DTNB (5,5-dithio-bis-(2-nitrobenzoic acid)), which reacts with CoA's free thiol group to yield a mixed disulfide and the yellow-colored TNB^2−^ (2-nitro-5-thiobenzoic acid).^32^ The concentration of TNB was then determined by measuring its absorbance at a wavelength of 412 nm. Tissue homogenate was thawed on ice just prior to the assay and diluted to 0.1 mg/mL with 50 mM KPi buffer supplemented with 0.05% decylubiquinone (DDM) and achieving a final concentration of 0.025% DDM; 80 µL assay buffer (50 mM KPi supplemented with 100 µM DTNB and 300 µM acetyl-CoA (final concentrations) was distributed into each well, and 100 µL of diluted homogenate was added to each well in triplicate. The enzymatic reaction commenced with the introduction of 20 µL oxaloacetate to a final concentration of 500 µM. Absorbance at 412 nm was then measured over a 10-minute period at room temperature in 7-second cycles using a SPECTRAmax Plus 384 plate reader (Molecular Devices, UK). The concentration of TNB^2−^ was calculated using the molar extinction coefficient of ε412 = 13 600 M^−1^cm^−1^.

### 2.13 Mitochondrial DNA damage assessment

This process involves measuring the extent of oxidative damage to mitochondrial DNA by using PCR with two different primer sets: one for a short segment of DNA [127 base pairs (bp)] to determine the number of mitochondria, and another for a longer segment (10 090 bp) where reduced amplification signals DNA damage.^24,33^ DNA was extracted from brain tissue with the DNeasy blood and tissue kit (QIAGEN), stored at −20 ˚C, and its concentration was later adjusted for the test. The PCR mix contained 200 pM dNTPs, 200 pM forward primer (Sigma, 5′-GCC AGC CTG ACC CAT AGC CAT AAT-3′), 200 pM reverse primer (Sigma, 5′− GCC GGC TGC GTA TTC TAC GTT A −3′ for short target, 5′-GAG AGA TTT TAT GGG TGT AAT GCG G-3′for long target) for both DNA targets, 100 ng/mL bovine serum albumin (New England Biolabs), and 1 mM magnesium acetate in buffer (TaKaRa). The reaction used 15 ng of DNA template, 35 μL PCR mastermix, and 1 U of LA Taq polymerase (TaKaRa), with separate tubes for duplicate reactions and controls. The amplification was carried out for the short DNA target: 94 ˚C for 3 min followed by 16 cycles of 94 ˚C for 30 s, 64 ˚C for 45 s, and 72 ˚C for 45 s, followed by 72 ˚C for 10 min. For the long target: 94 ˚C for 3 min, followed by 16 cycles of 94 ˚C for 15 s and 64 ˚C for 12 min, followed by 72 ˚C for 10 min on a PCR thermocycler (Biometra). Afterward, the size of the PCR products was checked via gel electrophoresis. Concentrations of the PCR products were determined by measuring the emission of the samples at 488 nm and excitation at 525 nm after the addition of Pico-Green (Thermo) at a 1:200 dilution. Amplification of the long product (>10 kb) normalized to amplification of the short template (∼100 bp) is used to assess the degree of mtDNA damage; more damage coincides with a decrease in amplification of the long template.^34^ Finally, the data were adjusted against controls and normalized for comparison in Excel.

### 2.14 Randomization and blinding

Animals were randomly allocated to their intervention group on the day of the experiment. Samples for mass spectrometry and oxidative damage analysis were randomized and analyzed blindly. Image files were randomized and blinded by an independent investigator to blind the investigator conducting the analysis.

### 2.15 Statistical analyses

For comparing multiple groups, a one-way analysis of variance (ANOVA) with Tukey’s *post hoc* test was used. For comparing multiple groups with more than one independent variable, an ANOVA with either Sidak or Tukey’s *post hoc* test was used. For comparing two unpaired groups, a two-tailed unpaired *t*-test was used. For comparing two paired groups, a Mann–Whitney test was used. The Pearson correlation coefficient was used to determine linear correlation, whereas the Spearman correlation coefficient was used to determine linear correlation in non-parametric groups. Data are presented as mean values ± SEM, median ± SEM, or individual data points. A *P-*value less than 0.05 was considered significant. All statistical analysis was carried out using GraphPad Prism version 10.

## 3. Results

To explore the feasibility of local administration of aDSM intra-arterially within the brain of a patient undergoing MT, we extended and adapted the established murine tMCAO model^17^ (*Figure 2A*). For this, we combined a polyimide-based catheter with an internal 6-0 silicone occlusion suture (see Supplementary Data Supplementary material online, Figure*S1A*). The occlusion suture is first inserted into the CCA and advanced to the MCA to bring about MCAO, as verified by measuring cerebral blood flow (CBF) by laser Doppler flowmetry (see Supplementary Data Supplementary material online, Figure*S1B*). Shortly prior to reperfusion, the infusion catheter is then inserted into the carotid artery (CA) on top of the occlusion suture and guided to the region of occlusion/ischemia.

After a period of ischemia, the suture can be withdrawn, mimicking the reperfusion that follows MT, while at the same time aDSM can be infused via the catheter, thereby administering aDSM directly to the ischemic region during the critical period at the onset of reperfusion (*Figure 2B*). Incorporation of the infusion catheter (*n* = 4) led to the same succinate accumulation within the ipsilateral hemisphere following ischemia compared with the suture alone (*n* = 4; see Supplementary Data Supplementary material online, Figure*S1C*). Furthermore, infusing saline through the catheter (*n* = 7) upon removal of the suture after 30-min ischemia did not affect brain infarct volume after 2 h reperfusion (see Supplementary Data Supplementary material online, Figure*S1D*), or CBF upon reperfusion when compared to suture alone (see Supplementary Data Supplementary material online, Figure*S1E*). Furthermore, local infusion of saline 2 min prior to reperfusion did not initiate early reperfusion. Therefore, incorporation of the catheter and local infusion of saline do not affect IRI, making this a viable model for assessing the therapeutic potential of aDSM in conjunction with MT (*Figures 1A, 2A*).

We chose aDSM as an adjunct neuroprotectant because of its far greater potency than neutral DSM in treating cardiac IRI, which is due to the elevated uptake of malonate via MCT1 at low pH (*Figure 1B*).^16^ MCT1 is widely expressed in the brain, with MCT1 predominating in the endothelial cells of the blood–brain barrier (BBB) while MCT2 and 4 are highly expressed in neurons and glial cells.^35–37^ Thus, aDSM should be far more potent in the brain than neutral DSM. Acidification of DSM at pH 6 enhanced brain uptake following administration via the tail vein with an infusion pump (100 µL; 50 µL/min) compared to pH 7.4 (*n* = 5 each)(see Supplementary Data Supplementary material online, Figure*S2A*). Furthermore, aDSM given via the tail vein (*n* = 6) was more protective against IRI measured 2 h after reperfusion in the tMCAO model than neutral DSM (*n* = 6; see Supplementary Data Figure 2*B*). Cerebral administration of DSM via the intra-arterial catheter [50 µL; 12.5 µL/min (*n* = 6)] greatly enhanced malonate uptake into the brain compared to administration via the tail vein (*n* = 5; see Supplementary Data Supplementary material online, Figure*S2C*), and the increased uptake due to local intra-arterial administration was further enhanced by lowering the pH to 6 (*n* = 6; see Supplementary Data Supplementary material online, Figure*S2C*). Uptake of aDSM following intra-arterial administration was decreased by the selective MCT1 inhibitor AR-C141990 given 5 min prior to aDSM administration (*n* = 5; see Supplementary Data Supplementary material online, Figure*S2C*), confirming the rapid uptake of aDSM through the BBB into the brain via MCT1. Furthermore, uptake of aDSM into aerobic brain tissue led to succinate accumulation (*n* = 3; see Supplementary Data Supplementary material online, Figure*S2D*), confirming aDSM entry into brain cell mitochondria and engagement with its target SDH. Finally, aDSM infused intra-arterially during reperfusion after ischemia was taken up into the brain (*n* = 3; see Supplementary Data Supplementary material online, Figure*S2E*).

**Figure 3:**
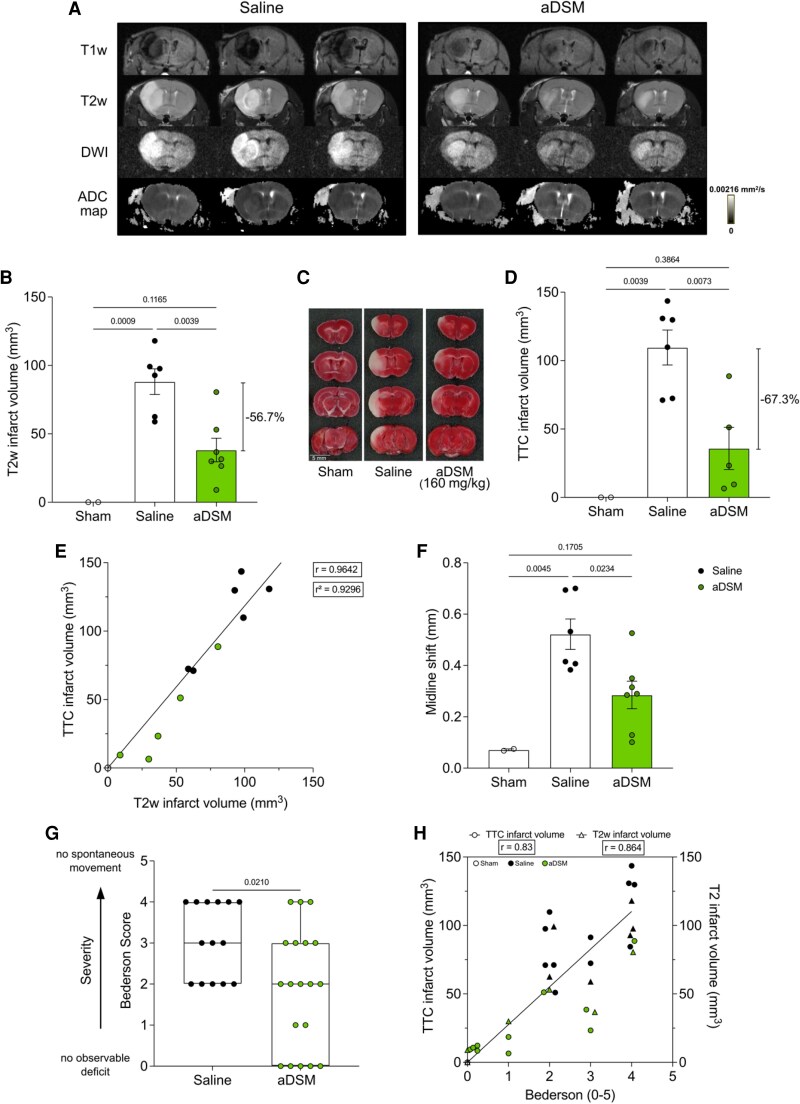
Infarct volume assessed by MRI and neurological function measured 24 h after reperfusion with aDSM. a: Representative MRI images (T2w, T1w, DWI, ADC map) measured 24 h after tMCAO followed by reperfusion with local infusion of saline or aDSM (160 mg/kg) (*n* = 3 in each group). b: Brain infarct volume obtained using T2w imaging 24 h following sham operation, or 24 h following tMCAO with local infusion of saline or aDSM (160 mg/kg; mean ± SEM, *n* = 2–7). Statistical significance was assessed by one-way ANOVA with Tukey’s *post hoc* test. *C*, *D*: Brain infarct volume (mm^3^) was measured by TTC staining at 24 h following sham operation, or 24 h following tMCAO with local infusion of saline or aDSM (160 mg/kg). *C* shows representative TTC-stained brain slices, and *D* shows full quantification (mean ± SEM, *n* = 2–6). Statistical significance was assessed by one-way ANOVA with Tukey’s *post hoc* test. *E*: Correlation of infarct volume (mm^3^) at 24 h following sham operation, or 24 h following tMCAO with local infusion of saline or aDSM (160 mg/kg). Correlation was assessed by Pearson correlation. *F*: Midline shift (mean ± SEM, *n* = 2–7). Statistical significance was assessed by one-way ANOVA with Tukey’s *post hoc* test. g: Bederson score assessed 24 h following tMCAO with local infusion of saline or aDSM (160 mg/kg). Data presented as median ± range, (*n* = 14–19). Statistical significance was assessed by two-tailed Mann–Whitney test. *H*: Correlation of Bederson score with infarct volume (mm^3^) measured by TTC staining (left hand y-axis) and by MRI T2w imaging (right-hand *y*-axis) at 24 h following tMCAO with local infusion of saline or aDSM (160 mg/kg). Correlation assessed by Spearman correlation.

The therapeutic potential of intra-arterial administration of aDSM from 2 min prior to 2 min after reperfusion onset was assessed by histology 2 h after the removal of the intraluminal filament (*Figure 2B*). There was a dose-dependent decrease in brain infarct volume, with an optimal aDSM dose of 160 mg/kg (n = 9; *Figure 2C*). Neither the slightly acidic pH or the osmolarity of the infusion contributed to protection, as was shown using saline at pH 6 (*n* = 5) or with disodium tartronate [160 mg/kg; pH 6 (*n* = 6)], structurally similar and osmotically identical to aDSM (see Supplementary Data Supplementary material online, Figure*S2F*). We next determined whether the protection against IRI by aDSM measured 2 h post reperfusion was sustained at the more clinically relevant time point of 24 h post-stroke using magnetic resonance imaging (MRI) at clinical field strength (3T), which is used to assess infarct volume and evolution in patients.^38–40^ The efficacy of aDSM (160 mg/kg) on MRI 24 h after reperfusion was assessed using anatomical T2-weighted (T2w) and T1-weighted (T1w) images, as well as diffusion-weighted imaging (DWI) and corresponding ADC maps (*Figure 3A*, Supplementary Data Supplementary material online, *Table S1*). Infusion of aDSM (*n* = 7) produced a statistically significant reduction of ∼57% in brain infarct volume from volumetric quantification using T2w MRI images compared to saline control (*n* = 6; *Figure 3B*). Protection against IRI at 24 h by aDSM was also seen when the infarct volume was assessed by TTC histology in both mm^3^ (*Figure 3C*) and % ipsilateral brain (see Supplementary data Supplementary material online, Figure*S3A*), with a statistically significant ∼67% reduction in infarct volume (*Figure 3D*). Infarct volume derived from T2w images correlated with that measured by TTC staining (Pearson correlation coefficient *r* = 0.9642; *Figure 3E*) and with those measured by DWI infarct volume (see Supplementary data Supplementary material online, Figure*S3B*). The decrease in infarct volume was accompanied by a significant reduction in vasogenic cerebral edema as measured by midline shift (*Figure 3F*), which also correlated with the infarct volume (see Supplementary data Supplementary material online, Figure*S3B*). Moreover, following aDSM treatment (*n* = 19), mice displayed improved neurological outcome determined by the Bederson score compared to saline-treated controls (*n* = 14) (*Figure 3G*). The additional mice in these experiments were from oxidative damage assay experiments, which could not be used to measure infarct volume, MRI, or TTC histology due to the incompatibility of the assays. Neurological outcome correlated with both MRI and TTC measures of infarct volume (*Figure 3H*). The loss in body weight typically found 24 h after reperfusion in the tMCAO model was unaffected by aDSM treatment (see Supplementary Data Supplementary material online, Figure*S3C*). We conclude that the local, intra-arterial administration of aDSM is neuroprotective when given upon reperfusion in our mouse model of MT.

**Figure 4:**
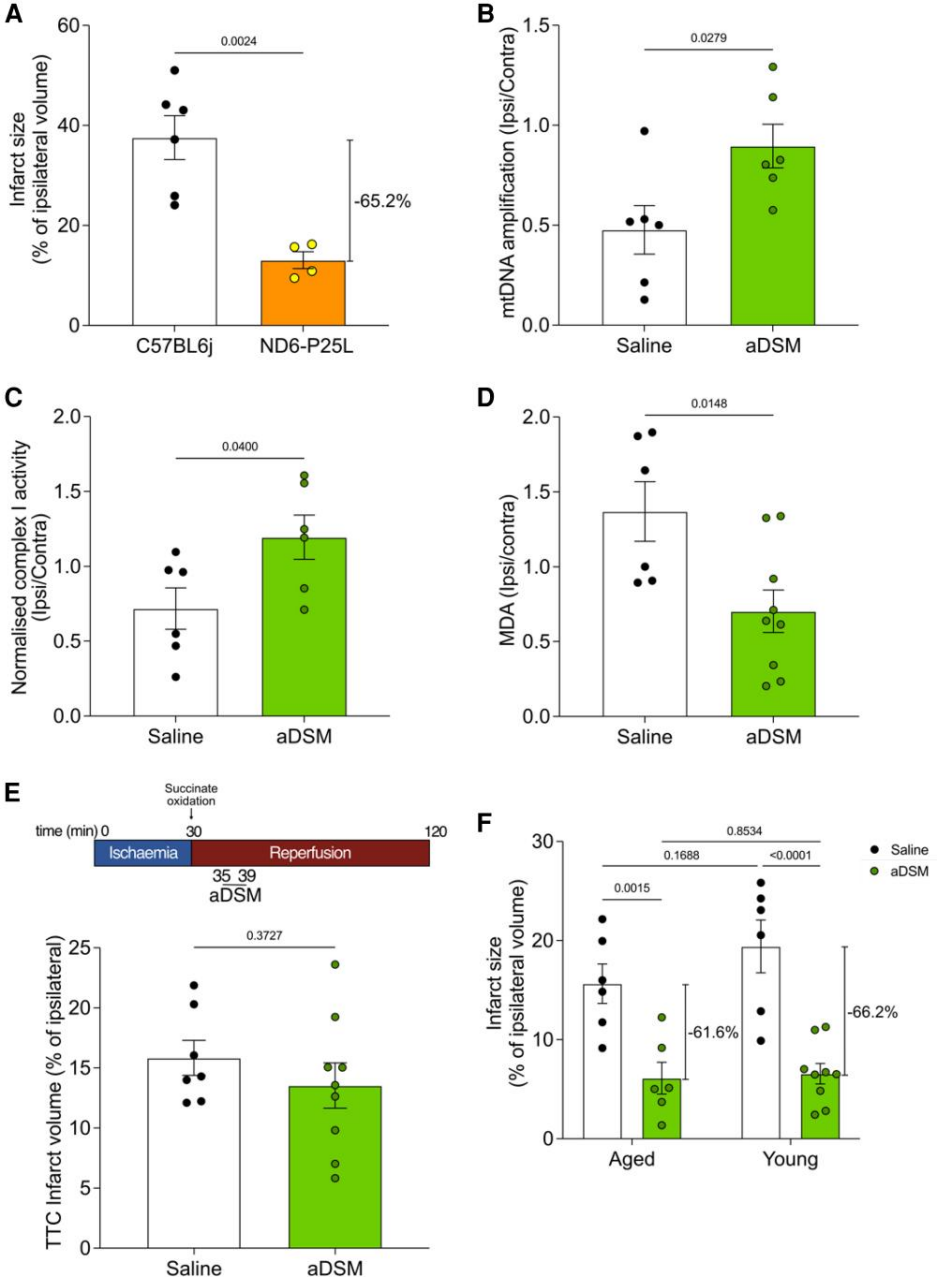
Intra-arterial aDSM prevents mitochondrial ROS production and oxidative damage following cerebral IRI. *A*: Brain infarct size of wild type or ND6-P25L mice assessed by TTC staining 24 h after tMCAO with local infusion of saline (mean ± SEM, *n* = 4–6). Statistical significance assessed by two-tailed unpaired Student’s *t*-test. *B*: Relative amplification of long and short mtDNA sections following tMCAO with local infusion of saline, or aDSM (160 mg/kg) at 24 h. Data are presented as the amplification in the ipsilateral hemisphere relative to that in the contralateral hemisphere (mean ± SEM, *n* = 6). Statistical significance was calculated by a two-tailed unpaired Student’s *t*-test. *C*: Complex I activity normalized to that of citrate synthase was determined 24 h after tMCAO with local infusion of saline, or aDSM (160 mg/kg). Data are presented as the normalized complex I activity in the ipsilateral hemisphere relative to that in the contralateral hemisphere (mean ± SEM, *n* = 6). Statistical significance was calculated by a two-tailed unpaired Student’s *t*-test. d: MDA was assessed 24 h after tMCAO with local infusion of saline, or aDSM (160 mg/kg). Data are presented as the levels in the ipsilateral hemisphere relative to that in the contralateral hemisphere (mean ± SEM, *n* = 6–9). Statistical significance was calculated by a two-tailed unpaired Student’s *t*-test. *E*: Brain infarct size was assessed by TTC staining 24 h after tMCAO followed 5 min later by local infusion of saline or aDSM (160 mg/kg; mean ± SEM, *n* = 7–9). Statistical significance was determined by a two-way ANOVA with Tukey’s *post hoc* test. *F*: Brain infarct size was assessed by TTC staining 2 h after tMCAO following local infusion of saline or aDSM (160 mg/kg) in young (8–12 weeks of age) and old (55–57 weeks of age) mice (mean ± SEM, *n* = 6–9). Statistical significance was determined by a two-way ANOVA with Tukey’s *post hoc* test.

The therapeutic effect of aDSM against cardiac IRI is due to acute SDH inhibition, preventing RET-ROS production via complex I upon reperfusion^41^ (*Figure 1B*). To determine if this is also how aDSM prevents cerebral IRI, we next used mice homoplasmic for the mtDNA G14600A mutation that leads to a P25L amino acid substitution in the ND6 subunit of complex I that prevents RET-ROS production and prevents cardiac IRI.^41^ When ND6-P25L mice (*n* = 4) were exposed to IRI following tMCAO, there was a statistically significant 65.2% decrease in infarct volume, measured by TTC staining 24 h after reperfusion compared to wild-type (WT) mice [*n* = 6 (*Figure 4A*)]. The RET-ROS production from complex I upon reperfusion is thought to disrupt cell viability by inducing mitochondrial oxidative damage. To assess if this also occurred during IRI in our model, we assessed cerebral oxidative damage 24 h after reperfusion. We first measured mtDNA damage by comparing the PCR amplification of mtDNA long and short amplicons, where decreased amplification of the long vs. short sections of mtDNA indicates damage caused by deletions or base adduct formation.^34^ IRI decreased mtDNA amplification (*n* = 6), and this damage was prevented by aDSM upon reperfusion (*n* = 6) (*Figure 4B*). Complex I activity is sensitive to mitochondrial oxidative damage by RET due to proximity to the ROS source^42^ and its activity, normalized to that of the mitochondrial matrix enzyme citrate synthase, was decreased by IRI in the ipsilateral compared to contralateral hemispheres (*n* = 6), and this damage was prevented following aDSM administration (*n* = 6) (*Figure 4C*, Supplementary Data Supplementary material online, Figure*S4B*). Finally, we assessed lipid peroxidation by quantifying MDA formation upon processing of ipsilateral vs. contralateral hemispheres of mouse brains 24 h after reperfusion. MDA Production *ex vivo* was increased following IRI, indicating elevated lipid peroxidation *in vivo* during IRI (*n* = 6), and this was decreased by aDSM (*n* = 9; *Figure 4D*, Supplementary Data Supplementary material online, Figure*S4C*). In the heart, IRI occurs in the first few minutes of reperfusion; thus, aDSM has to be administered at the onset of reperfusion to block this early ROS burst. To see if this was also the case in cerebral IRI, we infused aDSM (160 mg/kg, pH 6) into the ischemic region 5 min after reperfusion and found that this was not protective (*n* = 9; *Figure 4E*). Thus, the protection afforded by administration of aDSM upon reperfusion is due to the prevention of the initial ROS burst upon reperfusion from complex I by RET that leads to the oxidative damage that underlies IRI.

As stroke disproportionately affects the aged population with worse clinical outcome compared to younger patients,^43,44^ we assessed the efficacy of aDSM in old mice (55–57 weeks of age, equivalent to late middle age in humans) and found that old mice showed similar baseline (*n* = 6) IRI following reperfusion after ischemia and were equally protected by aDSM infusion (*n* = 6) as young (8–12 weeks of age) mice (*Figure 4F*). Furthermore, stroke patients often present with comorbidities;^45,46^ therefore, we also assessed the efficacy of aDSM in diabetic and phenotypically obese db/db mice. Db/db mice exhibited aggravated damage with a higher baseline infarct size (*n* = 4), which was similarly protected by aDSM infusion (*n* = 6; see Supplementary Data Supplementary material online, Figure*S5*). As thrombolytics such as rtPA are often given systemically to ischemic stroke patients in combination with MT, we next assessed if there were any drug-drug interactions between aDSM and rtPA (see Supplementary Data Supplementary material online, Figure*S6*). In mice treated with rtPA (*n* = 5) mirroring that of patients in clinic (0.9 mg/kg, 10% bolus 90% intravenously over 60 min),^47,48^ there was no effect on the efficacy of aDSM in decreasing IRI (see Supplementary Data Supplementary material online, Figure*S6A*). There was also no effect of aDSM on rtPA protease activity *in vitro* (see Supplementary Data Supplementary material online, Figure*S6B*–*E*).

In mouse models of tMCAO, 30 min of ischemia is widely used to generate a severe infarct.^49–54^ Even so, we also assessed the efficacy of aDSM in a longer 60-min tMCAO model. Mice subjected to 60-min ischemia and 24-h reperfusion (*n* = 6) did not have a significant increase in infarct volume compared to the 30-min ischemia (*n* = 6) in the saline controls (see Supplementary Data Supplementary material online, Figure*S7*).

## 4. Discussion

MT has emerged as the standard of care for the treatment of medium and large vessel ischemic stroke.^55^ Even so, the outcome for many patients after MT is still poor due to the IRI associated with reperfusion. Consequently, neuroprotective therapies that decrease this IRI have been long sought, but the outcomes of candidate drugs in Phase III trials have been disappointing.^11,56,57^ Recently, aDSM has emerged as an attractive candidate neuroprotectant. Here, we tested this possibility by extending a mouse model of tMCAO in order to mimic intra-arterial infusion of aDSM during treatment of ischemic stroke by MT.

We showed that the local, intra-arterial administration of aDSM led to far greater tissue uptake of malonate than peripheral administration of aDSM or local infusion of DSM. Furthermore, this uptake through the BBB occurred via MCT1, and within brain cells, engaged with its target, SDH. Most importantly, locally administered aDSM was far more potent in protecting the brain against IRI than intravenous treatment. In assessing efficacy, we quantified the decrease in infarct volume using clinically relevant T2w MRI images at 24 h post reperfusion, which correlated with preservation of functional neurological outcome. Interestingly, the maximal decrease in infarct volume post IRI upon administration of aDSM was about 60%, which matched the decrease in infarct volume found in the ND6-P25L mice compared to WT mice. As complex I in the ND6-P25L mice cannot produce ROS by RET, this is consistent with the hypothesis that preventing succinate oxidation by aDSM acts by preventing RET-ROS, and that the maximum possible decrease in infarct volume by preventing the IRI damage due to succinate oxidation with aDSM is ∼ 60%.

Aged and diabetic obese db/db mice were also protected against stroke IRI by aDSM, an important consideration from a clinical perspective. We acknowledge that the lack of female mice is a limitation to this study, though we hope to comprehensively characterize the uptake and efficacy of aDSM in female mice in the future.

Interestingly, extended ischemic insult (60 min vs. 30 min) did not result in larger infarct volumes, but did, however, ameliorate the efficacy of aDSM. The comparable infarcts may be due to the use of the 6–0 occlusion suture in this study, which although commonly used, is larger than the 7–0 and 8–0 occlusion sutures also used in some mouse models of tMCAO. Furthermore, the incorporation of the infusion catheter may increase the chance of arterial spasms or embolisms from clot formation at the catheter tip, which may result in larger infarcts from 30 min of ischemia. Regarding the lack of efficacy from aDSM after 60-min ischemia, a likely explanation is that a smaller salvageable penumbra is present in the brain following 60-min ischemia vs. 30 min as more brain tissue became irreversibly damaged, and therefore, not amenable to protection with aDSM. It is important to consider that in human MT, patient selection is not only dependent on the time since symptom onset, but also on the presence of a salvageable penumbra. Multiple trials show the efficacy of MT in early thrombectomy window (∼ < 6 h), whereas those that show efficacy in the later window (up to 24 h) utilized advanced imaging techniques such as MRI and CT perfusion to evaluate the size of the ischemic core and viable penumbra for patient selection.^7,8,58^ Therefore, it follows that patients who meet the criteria for MT are also those who could benefit from aDSM.

In summary, here we have developed a mouse model of administering a neuroprotective agent upon reperfusion during MT for ischemic stroke. Using this model, we show that intra-arterial administration of aDSM during MT attenuates IRI, supporting the translation of aDSM as an adjunct therapy for MT to improve clinical outcomes for ischemic stroke patients.

## Supplementary Material

cvaf118_Supplementary_Data

## Data Availability

The data underlying this article will be shared on reasonable request to the corresponding author.
