# Supplementary material for: Local arterial administration of acidified malonate as an adjunct therapy to mechanical thrombectomy in ischemic stroke
Source: Cardiovasc Res. 2025 Jun 27;121(9):1407–18. doi: 10.1093/cvr/cvaf118 (PMC12352302; doi:10.1093/cvr/cvaf118)
Supplement: cvaf118_Supplementary_Data [file cvaf118_supplementary_data.docx]

**Supplementary Material**

**Tables**

**Supplementary Data Table 1**. MRI sequence parameters

| **Parameter** | **T1 RARE**  **inversion-recovery** | **T2 Turbo RARE** | **DWI-SE** | **DWI-SE-EPI** |
| --- | --- | --- | --- | --- |
| Repetition Time  (ms) | 3000 | 2500 | 2000 | 1500 |
| Echo Time  (ms) | 13 | 60 | 60 | 35 |
| Inversion time  (ms) | 850 | - | - | - |
| Flip angle  (degrees) | 90 | 90 | 90 | 90 |
| Number of Signal  Averages | 5 | 14 | 2 | 28 |
| Field of View  (mm^2^) | 20 × 20 | 20 × 20 | 18 × 18 | 18 × 18 |
| Matrix size | 128 × 128 | 224 × 224 | 128 × 128 | 128 × 128 |
| Bandwidth (kHz) | 158.73 |  | 208.33 | 170.66 |
| Resolution (mm^2^) | 0.156 × 0.156 | 0.089 ×  0.089 | 0.141 × 0.141 | 0.141 × 0.141 |
| Slice thickness (mm) | 0.80 | 0.80 | 1 | 1 |
| Number of slices | 14 | 14 | 14 | 14 |
| RARE factor | 2 | 8 | - | - |
| Echo spacing (ms) | - | 15 | - | - |
| No. of echoes | - | - | - | - |
| No. b0 images | - | - | 1 | 2 |
| No. of diffusion directions |  | - | 1 | 1 |
| b-values (s/mm^2^) | - | - | 1800 | 200, 400, 600, 800, 1000, 1200 |
| Gradient duration/  separation (ms) | - | - | 2.5/10 | 2.5/10 |
| Scan time (mm:sec) | 11:30 | 16:20 | 12:48 | 14:42 |

Abbreviations: ADC; apparent diffusion coefficient; DWI, diffusion-weighted imaging; DWI-SE, diffusion-weighed spin-echo; RARE, rapid acquisition relaxation enhancement; SE-EPI, spin-echo echo-planar imaging

**Supplementary Data
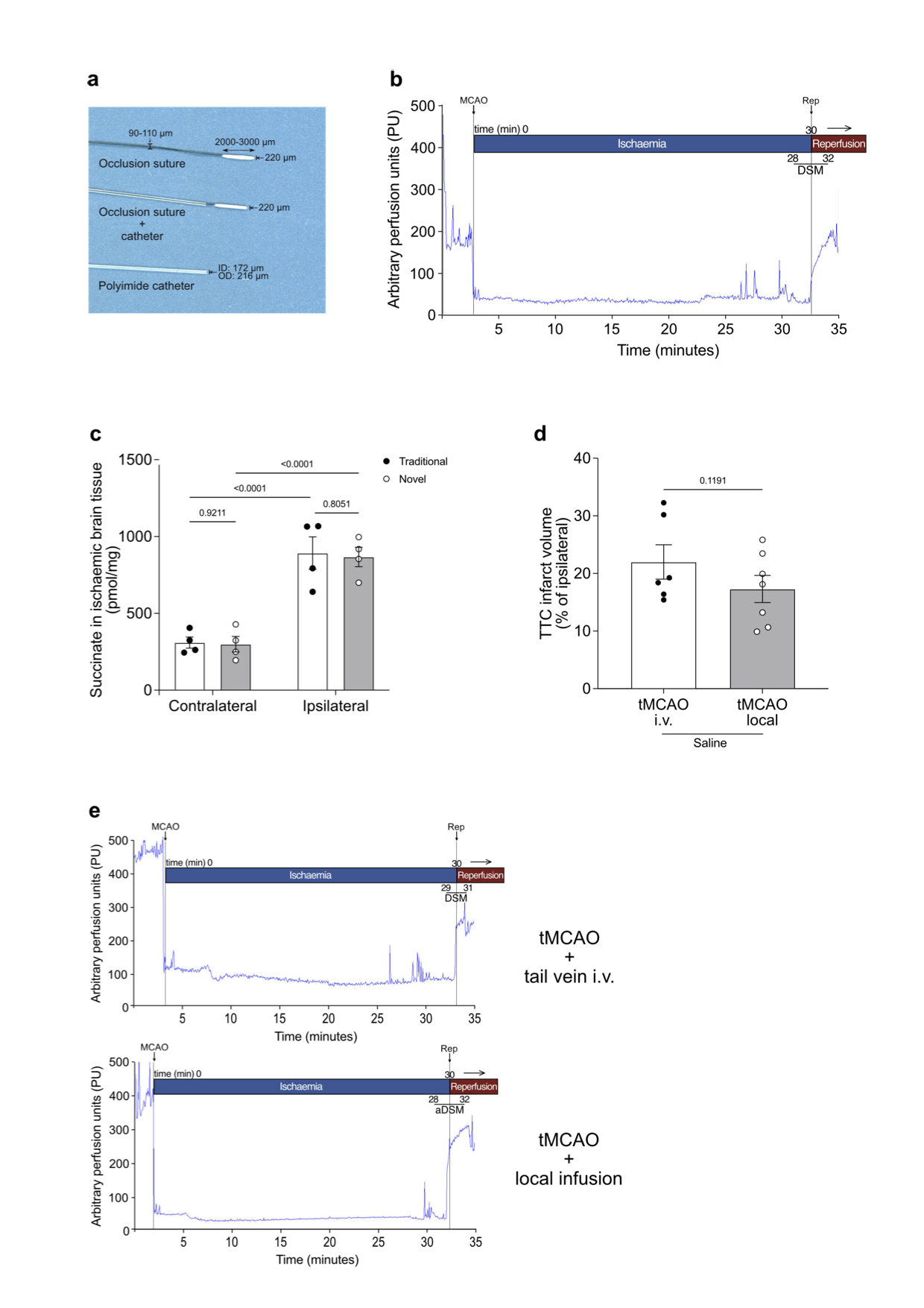
** **Supplementary Data Fig.1| Development of a murine tMCAO model in conjunction with local intra-arterial infusion of aDSM. a**: Images of the occlusion suture, the infusion catheter, and the occlusion suture inserted within the infusion catheter. **b**: Representative laser Doppler flowmetry trace of CBF in the MCA territory, showing sequential blockage of CA blood flow by insertion of the catheter, followed by MCAO by insertion of the occlusion suture. After a period of ischaemia the suture was withdrawn from the MCA to the CA leading to restoration of blood flow. **c**: brain succinate levels were assessed by LC-MS/MS in the ipsilateral and contralateral brain hemispheres after 30 min tMCAO (mean ± SEM, n = 4). Statistical significance was assessed by a two-way ANOVA with Sidak post-hoc test. **d**: Brain infarct size was measured by TTC staining at 2 h following tMCAO alone with intravenous infusion of saline (100 µl) starting 1 min before reperfusion and continuing for 2 min, or tMCAO with local infusion of saline (50 µl) starting 2 min before reperfusion and continuing for 4 min (mean ± SEM, n = 6 - 7). Statistical significance was determined by a two-tailed unpaired Student’s t-test. **e**: Representative laser Doppler flowmetry trace of CBF in the MCA territory showing sequential blockage of CA blood flow by insertion of the catheter, followed by MCAO by insertion of the occlusion suture. After a period of ischaemia the suture was withdrawn from the MCA to the CA leading to restoration of blood flow with either intravenous or local infusion.

**
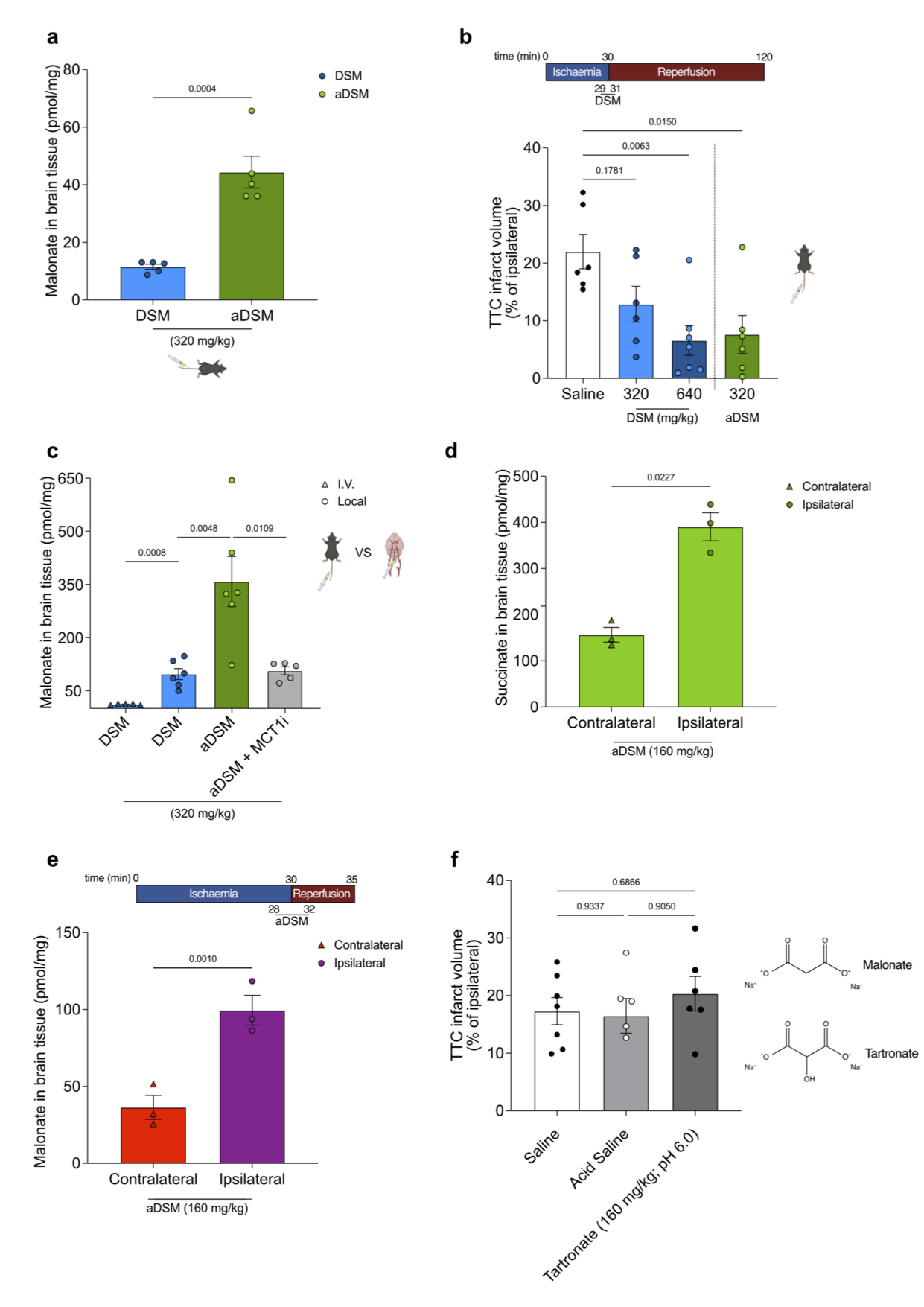
**

**Supplementary Data Fig.2| Local administration of acidified malonate improves malonate potency against IRI in tMCAO. a**: Brain tissue malonate levels assessed 5 min after tail vein i.v. administration of 320 mg/kg DSM or aDSM (pH 6.0) (mean ± SEM, n = 5). Statistical significance was assessed by a two-tailed unpaired Student’s t-test. **b**: Brain infarct size was measured by TTC staining at 2 h following tMCAO with infusion of saline, DSM, or aDSM (100 µl) via the tail vein starting 1 min before reperfusion and continuing for 2 min. Statistical significance was determined by a one-way ANOVA with Tukey post hoc test (mean ± SEM, n = 6-7). **c**: Brain tissue malonate levels assessed 5 min after either tail vein i.v. administration or local intra-arterial administration of 320 mg/kg DSM or aDSM, or aDSM in which mice were pre-treated with MCT1 inhibitor AR-C144190 locally 5 mins prior malonate delivery (mean ± SEM, n = 5-6). Statistical significance was assessed by a two-tailed unpaired Student’s t-test. d: Brain succinate levels in both brain hemispheres were assessed 5 mins after local intra-arterial administration of 160 mg/kg aDSM (mean ± SEM, n = 3). Statistical significance was assessed by a two-tailed paired Student’s t-test. **e**: Brain malonate levels were measured in both hemispheres following tMCAO followed by local infusion of aDSM (160 mg/kg) (50 µl) starting 2 min before reperfusion and continuing for 4 min, with brain being sampled at 5 min post reperfusion. (mean ± SEM, n = 3). Statistics: Two-tailed paired Student’s t-test. **f:** Brain infarct size was measured by TTC staining at 2 h following tMCAO with local infusion of saline, acidified saline (pH 6.0) or disodium tartronate (160 mg/kg; pH 6.0) (50 µl) starting 2 min before reperfusion and continuing for 4 min (mean ± SEM, n = 5-7). Statistics: One-way ANOVA with Tukey post hoc test.

**
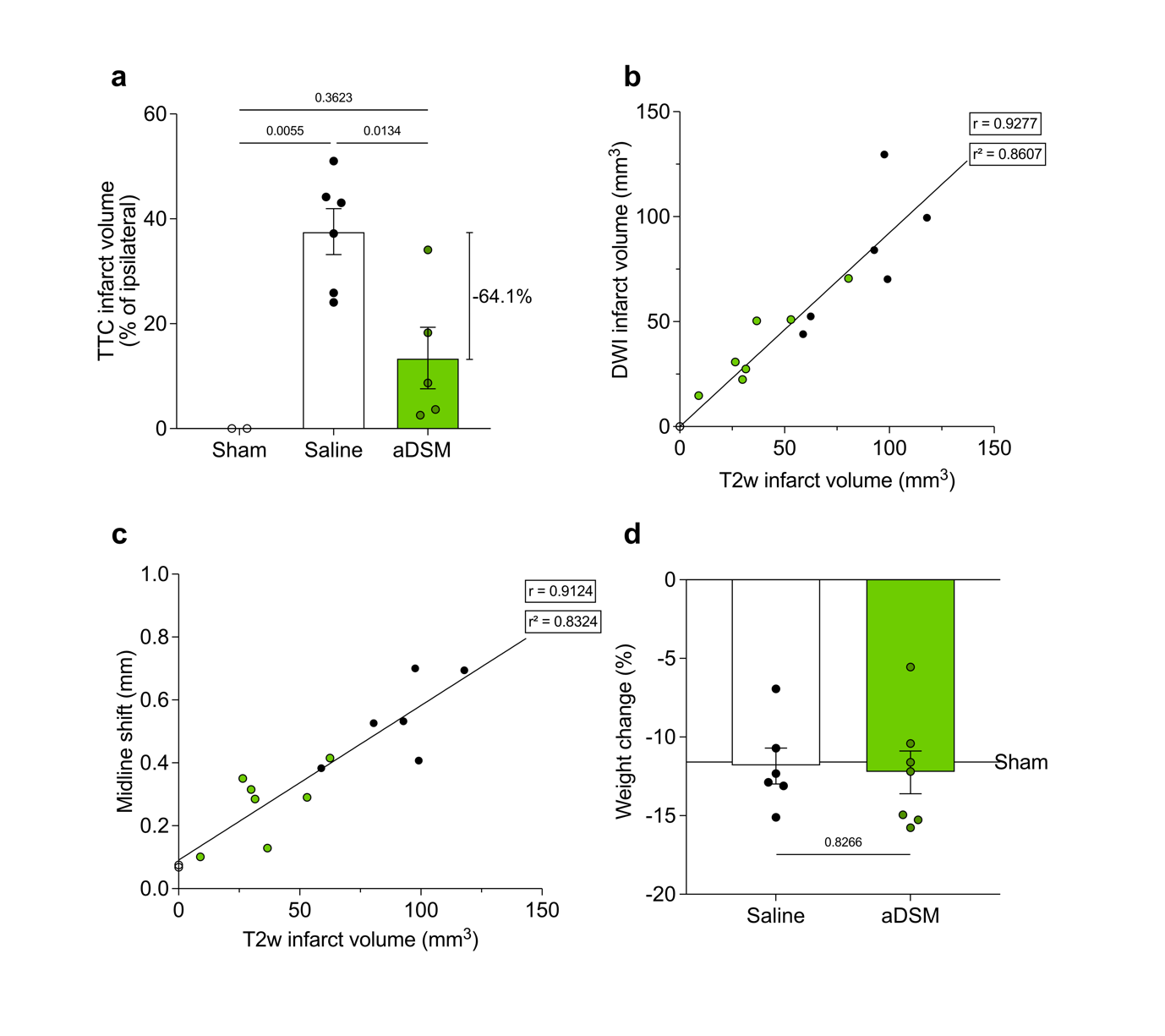
**

**Supplementary Data Fig.3| Locally delivered acidified malonate upon assessed at 24 hours. a:** Brain infarct volume (% ipsilateral) was measured by TTC staining at 24 h following sham operation, or 24 h following tMCAO with local infusion of saline or aDSM (160 mg/kg). **b:** Correlation of DWI and T2w derived infarct volume at 24 h following sham operation, or 24 h following tMCAO with local infusion of saline or aDSM (160 mg/kg). Correlation assessed by Pearson correlation. **c:** Correlation of midline shift and T2w infarct volume at 24 h following sham operation, or 24 h following tMCAO with local infusion of saline or aDSM (160 mg/kg). Correlation assessed by Pearson correlation. **d**: Percentage weight loss from prior tMCAO to that measured 24 h after reperfusion with local infusion of saline or aDSM (160 mg/kg) (mean ± SEM, n = 6-7). Statistics: Two-tailed unpaired t-test.

**
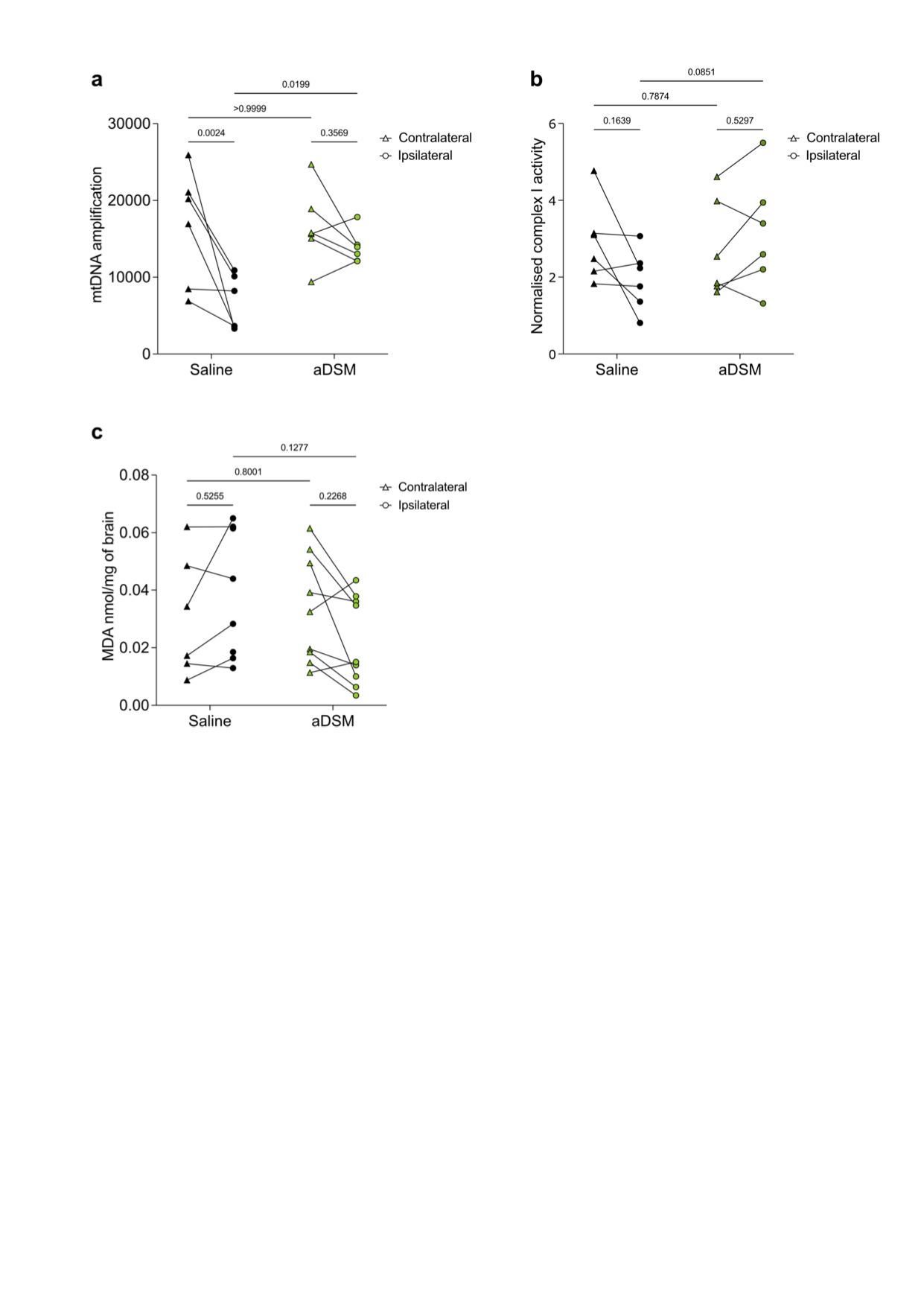
**

**Supplementary Data Fig.4| Acidified malonate is protective against oxidative damage during IRI. a:** Relative amplification of long and short mtDNA sections following tMCAO with local infusion of saline, or aDSM (160 mg/kg) at 24 hours. Data are presented as the amplification in the ipsilateral hemisphere relative to that in the corresponding contralateral hemisphere (n = 6 per group). Statistical significance was calculated by Two-way ANOVA with Sidak post-hoc test. **b**: Complex I activity normalised to that of citrate synthase was determined 24 h after tMCAO with local infusion of saline, or aDSM (160 mg/kg). Data are presented as the normalised complex I activity in the ipsilateral hemisphere relative to that in the corresponding contralateral hemisphere (n = 6 per group). Statistics: Two-way ANOVA with Sidak post-hoc test. **c**: MDA was assessed 24 h after tMCAO with local infusion of saline, or aDSM (160 mg/kg). Data are presented as the levels in the ipsilateral hemisphere linked to that in the corresponding contralateral hemisphere (mean ± SEM, n = 6-9). Statistics: Two-way ANOVA with Sidak post-hoc test.

**
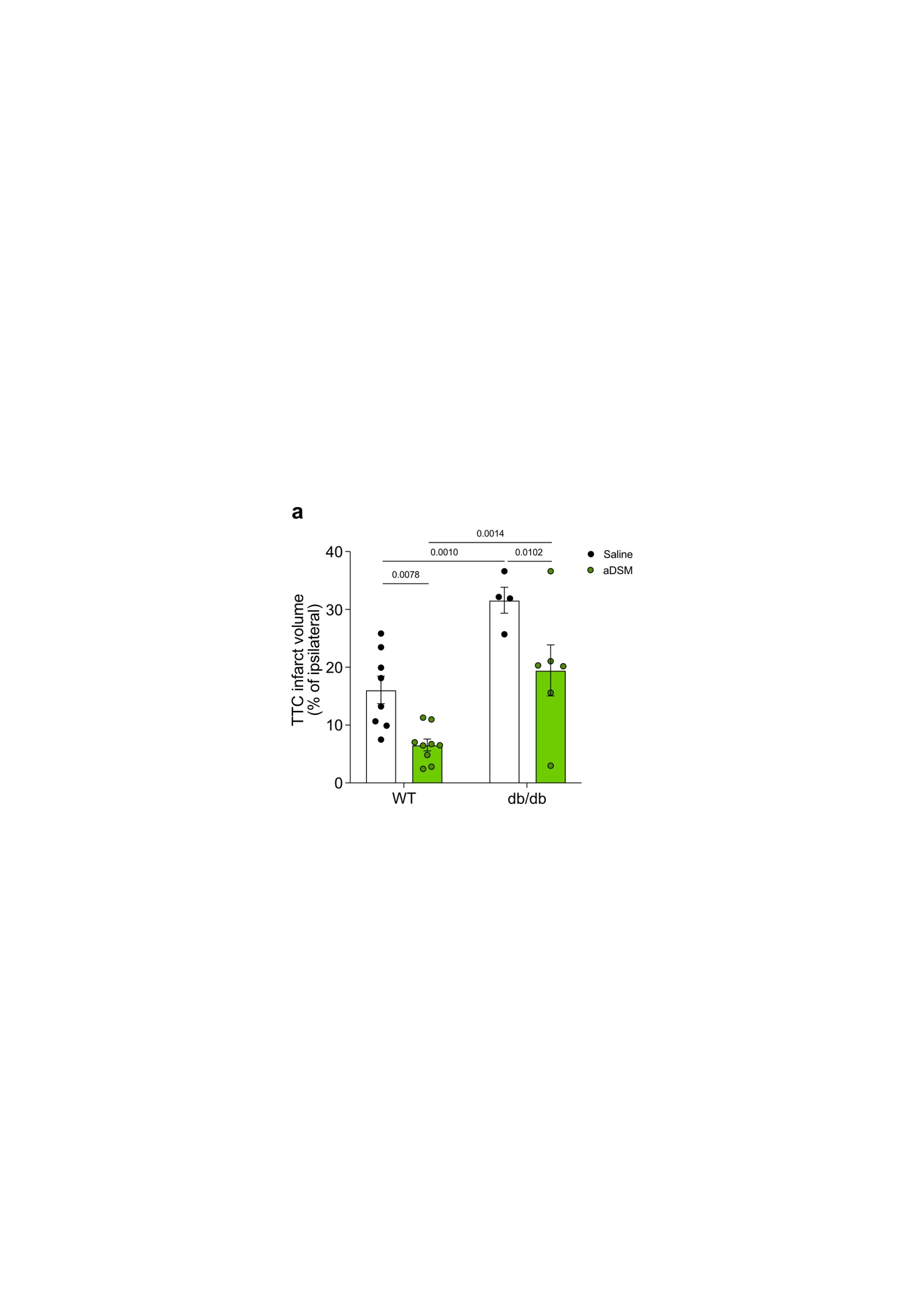
**

**Supplementary Data Fig.5| Neuroprotective effects of acidified malonate is preserved in diabetic mice. a**: Brain infarct size was measured by TTC staining at 2 h following tMCAO with local infusion of saline, or aDSM (160 mg/kg) (mean ± SEM, n = 4-9). Statistics: Two-way ANOVA with Sidak post hoc test.

**
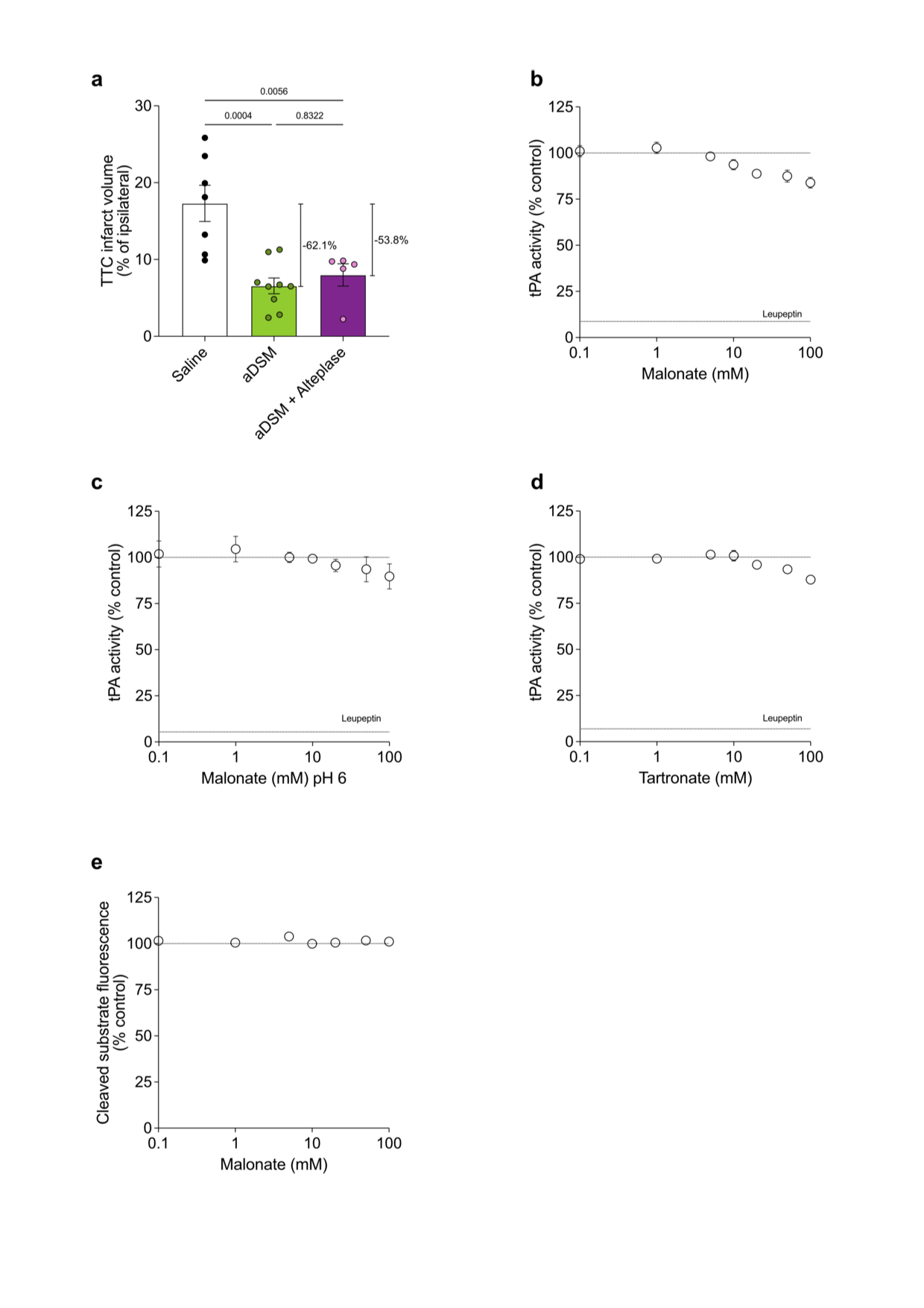
Supplementary Data Fig.6| Lack of drug-drug interactions between aDSM and rtPA. a**: Brain infarct size was measured by TTC staining at 2 h following tMCAO with local intra-arterial infusion (50 µl) starting 2 min before reperfusion and continuing for 4 min of saline, or aDSM (160 mg/kg) with or without intravenous rtPA treatment (0.9 mg/kg, 10% bolus 90% infusion over 60 mins) 2 mins before reperfusion (mean ± SEM, n = 5-9). Statistics: One-way ANOVA with Tukey post hoc test. **b, c**: Indicated concentrations of DSM or aDSM were incubated with the AMC tPA substrate before adding tPA and incubating at RT for 30 min then measuring AMC fluorescence. Data are mean ± SEM of 3 technical replicates. pH of solution at the end of the experiment was unchanged, even with high concentrations of malonate. **d**: Disodium tartronate at the indicated concentrations at pH 6 Was incubated with the AMC tPA substrate before adding tPA and incubating at RT for 30 min then measuring AMC fluorescence. Data are mean ± SEM of 3 technical replicates. **e:** tPA was added to AMC tPA substrate and incubated at RT for 30 min. Then indicated concentrations of malonate were added and AMC fluorescence measured. Data are presented as mean ± SEM of 3 technical replicates.


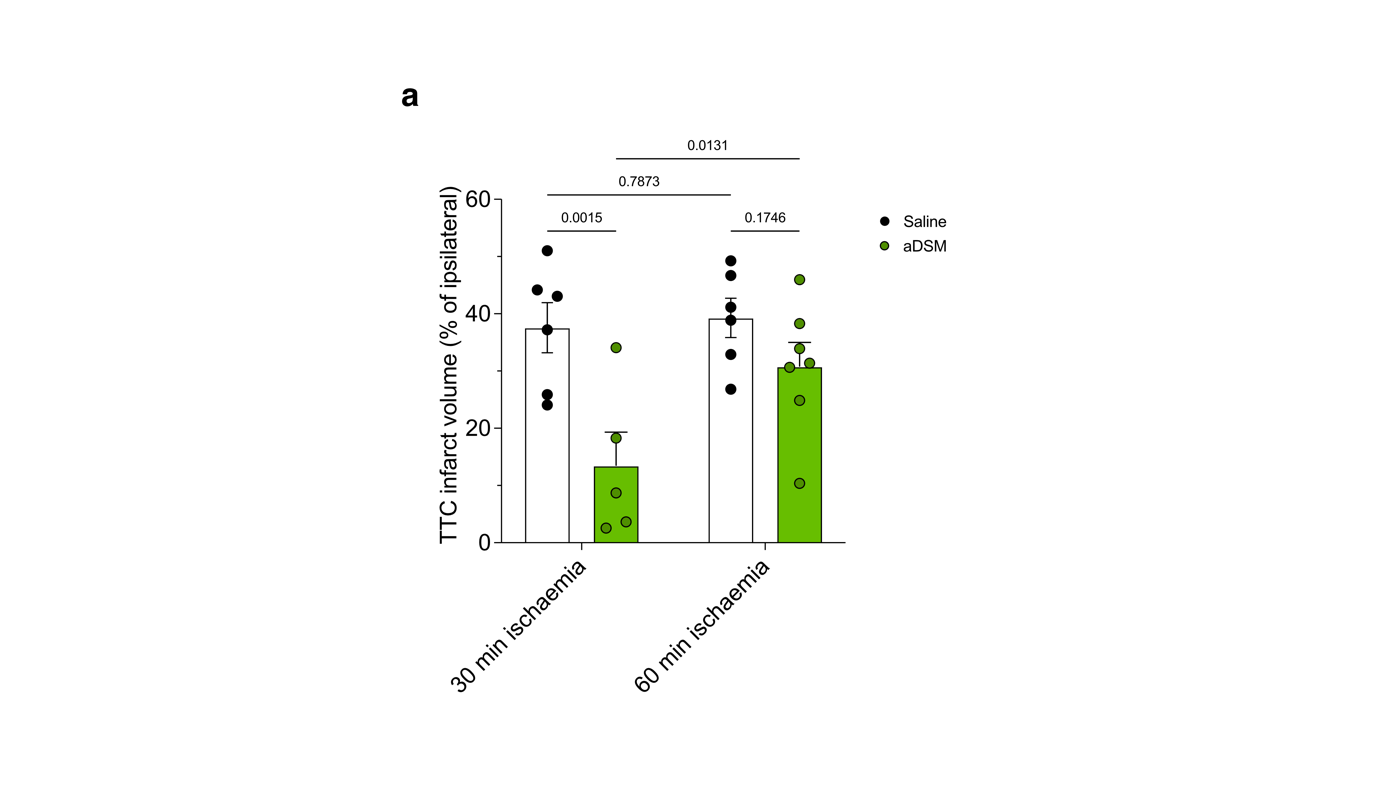


**Supplementary Data Fig.7| Efficacy of aDSM is lost following extended (60 mins) ischaemia a**: Brain infarct size was measured by TTC staining at 24 h following either 30 mins or 60 mins tMCAO with local intra-arterial infusion (50 µl) starting 2 min before reperfusion and continuing for 4 min of saline, or aDSM (160 mg/kg) 2 mins before reperfusion (mean ± SEM, n = 5-7). Statistics: Two-way ANOVA with Tukey post hoc test.
